# Homoeologous Chromosomes From Two *Hordeum* Species Can Recognize and Associate During Meiosis in Wheat in the Presence of the *Ph1* Locus

**DOI:** 10.3389/fpls.2018.00585

**Published:** 2018-05-01

**Authors:** María C. Calderón, María-Dolores Rey, Antonio Martín, Pilar Prieto

**Affiliations:** ^1^Plant Breeding Department, Institute for Sustainable Agriculture, Agencia Estatal Consejo Superior de Investigaciones Científicas (CSIC), Córdoba, Spain; ^2^John Innes Centre, Norwich Research Park, Norwich, United Kingdom

**Keywords:** wheat, barley, homoeologous pairing, introgressions, meiosis, chromosome recognition

## Abstract

Understanding the system of a basic eukaryotic cellular mechanism like meiosis is of fundamental importance in plant biology. Moreover, it is also of great strategic interest in plant breeding since unzipping the mechanism of chromosome specificity/pairing during meiosis will allow its manipulation to introduce genetic variability from related species into a crop. The success of meiosis in a polyploid like wheat strongly depends on regular pairing of homologous (identical) chromosomes and recombination, processes mainly controlled by the *Ph1* locus. This means that pairing and recombination of related chromosomes rarely occur in the presence of this locus, making difficult wheat breeding trough the incorporation of genetic variability from related species. In this work, we show that wild and cultivated barley chromosomes associate in the wheat background even in the presence of the *Ph1* locus. We have developed double monosomic wheat lines carrying two chromosomes from two barley species for the same and different homoeology chromosome group, respectively. Genetic *in situ* hybridization revealed that homoeologous *Hordeum* chromosomes recognize each other and pair during early meiosis in wheat. However, crossing over does not occur at any time and they remained always as univalents during meiosis metaphase I. Our results suggest that the *Ph1* locus does not prevent chromosome recognition and pairing but crossing over between homoeologous. The role of subtelomeres in chromosome recognition is also discussed.

## Introduction

More than two-thirds of global cropland features annual grain crops, which represent roughly 70% of humanity's food energy needs and typically grown in monoculture. Annual grain production, at its current scale, is fundamentally unsustainable. Thus, the growing human population demands greater crops, more productive and better adapted to specific agro-climatic conditions (Godfray et al., 2010). Plant breeders are playing a major role in worldwide efforts to understand gene functions and interactions with the aim of increasing quality and productivity of major crops. Wide-crossing in plant breeding is an important tool and sometimes the results are the starting point for new crops (Omara, 1953). For example, wide-crossing has been carried out in the Triticeae tribe, which includes wheat, to develop new plant species such as × *Triticosecale*, obtained after crossing wheat and rye, or × *Tritordeum*, an amphyploid between the wild barley *Hordeum chilense* Roem. et Schult. and wheat (Omara, 1953; Martín and Sanchez-Monge Laguna, 1982). Breeders have also used related species as genetic donors for widening the genetic basis of wheat to get for example wheat cultivars better adapted to specific agro-climatic conditions, improving the quality or carrying resistance to diseases (Lukaszewski, 2000; Liu et al., 2005; Calderón et al., 2012; Rey et al., 2015a). In fact, there are many wild species carrying interesting traits that would be useful to be exploited in wheat breeding programmes, but unfortunately, hybridization between wheat and a wild related species produces only a low level of chromosome pairing and recombination. So understanding wheat genetics and genome organization is essential for plant breeding purposes.

Bread wheat is an hexaploid, which possesses three sets of related chromosomes because of doubling of chromosomes following sexual hybridization between closely related species. However, chromosomes associate regularly in pairs in wheat during meiosis, the cellular process to produce gametes in sexually reproducing organisms. Thus, at meiosis each chromosome only recognize and associate with its homologous and not with the related (homoeologous) chromosomes, which have a similar gene content and order but differ in repetitive DNA sequences. Several pairing homologous (*Ph*) genes control chromosome associations in wheat, although the major effect is due to the *Ph1* locus (Sears, 1976). The efficiency of chromosome associations during meiosis have a big influence on the fertility of wheat plants, being crucial for success in breeding, but has a negative effect preventing pairing and recombination between wheat chromosomes and those from related species. Therefore, it seems reasonable to go deeper into the knowledge of the biology of chromosome associations during meiosis in wheat, which will be valuable for wheat breeding.

Chromosome dynamics during meiosis have been extensively studied in a polyploidy such as hexaploid wheat (Moore, 2002; Corredor et al., 2007; Colas et al., 2008; Naranjo and Corredor, 2008). It is now well established that both interactions during recombination at the DNA level and assembly of a meiosis-specific proteinaceous structure known as the synaptonemal complex (SC) play roles in stabilizing associations between homologous chromosomes. However, how homologs became colocalized and how initial recognition is accomplished to establish chromosome associations remains poorly understood. When a chromosome recognizes its homolog (and not another chromosome) in wheat, a localized conformational change in adjacent chromatin is triggered in both partners. This process facilitates recognition and association of homologous versus homoeologous chromosomes and is affected by the *Ph1* locus (Prieto et al., 2005; Greer et al., 2012). Thus, *Ph1* stabilizes wheat during meiosis by both, promoting homolog synapsis during early meiosis and preventing homoeologous recombination later in meiosis (Martín et al., 2014, 2017). The effect on synapsis occurs during the telomere bouquet *Ph1* stage, when promotes more efficient homologous synapsis, thereby reducing the chance of homoeologous synapsis (Martín et al., 2017). The effect on CO formation occurs later in meiosis, when *Ph1* prevents MLH1 sites (Double Holliday Junctions marked to become COs) on synapsed homoeologues from becoming COs. In addition, it has been also described that the level of a ZIP4 paralog included within the *Ph1* locus alters the number of CO between homoeologous chromosomes (Rey et al., 2017).

Efforts focused on centromeres and telomeres behavior during meiosis have been also made (Martinez-Perez et al., 2000, 2001, 2003; Naranjo et al., 2005). Telomeres, which are highly conserved structures among plants, including wheat (Simpson et al., 1990; Ganal et al., 1991; Schwarzacher and Heslop-Harrison, 1991), play an important role on initial chromosome associations at the onset of meiosis. In this stage, the association of telomeres in a bouquet facilitates the search and recognition of homologous chromosomes by bringing chromosomes closer (Corredor and Naranjo, 2007; Koszul et al., 2008; Moore and Shaw, 2009) and its formation is affected by the *Ph1* locus (Richards et al., 2012). Subtelomeres, which are the telomere associated sequences (TAS), are highly polymorphic and extraordinarily dynamic sequences (Eichler and Sankoff, 2003). The complex and variable nature of subtelomeres has made difficult to assess the possible functions(s) of these regions so far, but studies on *Arabidopsis* and *Hordeum* subtelomeres might suggest a possible role on chromosome specificity between homolog chromosomes at the onset of meiosis (Kotani et al., 1999; Heacock et al., 2004; Calderón et al., 2014). In fact, subtelomeres in *Hordeum* showed high variability not only from different chromosomes but also among chromosome arms within the same chromosome (Schubert et al., 1998; Prieto et al., 2004b). Thus, the copy number of the subtelomeric HvT01 sequence was variable among chromosomes in both *H. chilense* and *H. vulgare*. Since chromosome associations are initiated at the distal regions of the chromosomes and homologous chromosomes are zipping up from those to the centromeres (Prieto et al., 2004a; Corredor et al., 2007), it seems reasonable to go deeper into the role of the subtelomeric regions on homolog chromosome associations, rather than focusing on features that are common to all chromosomes like telomeres.

The addition of a pair of “alien” chromosomes to the full genome complement of a crop species is commonly used as a first step for accessing genetic variation from the secondary gene pool, but addition lines are also relevant for understanding meiotic pairing behavior and chromosome structure (Friebe et al., 2005; Lukaszewski, 2010). Sets of both cultivated (*Hordeum vulgare*) and wild (*H. chilense*) barley addition lines in a hexaploid wheat background were developed (Islam et al., 1978, 1981; Miller et al., 1982) and have potential in plant meiosis studies. Certainly, it allows tracking one specific pair of chromosomes or chromosome segments within the wheat background using genomic *in situ* hybridization (GISH) and study chromosome rearrangements and associations exclusively in a pair of homologs (Naranjo et al., 2010; Rey et al., 2015b).

In this study, we have developed double monosomic addition lines of wild and cultivated barley in wheat for the same and for different homoeology group to go deeper into the knowledge of chromosome associations during meiosis. These double monosomic addition lines enabled to distinguish chromosomes from two different barley species in the wheat background, observe conformational changes during meiosis and analyze whether subtelomeres might play a role on chromosome recognition/pairing at early meiosis in the absence of homologs. Results showed that homoeologous chromosomes can recognize each other to associate correctly in pairs, even in the presence of the *Ph1* locus, although crossing over does not occur as they remained as univalents during metaphase I.

## Materials and methods

### Plant material

Crosses between *H. chilense* and *H. vulgare* addition lines in bread wheat (*Triticum aestivum* cv. Chinese Spring; AABBDD + pair of H^ch^H^ch^ and AABBDD + pair of H^v^H^v^, respectively) for the same and for different homoeology group were made to obtain double monosomic wheat lines carrying one *H. chilense* and one *H. vulgare* chromosome for the same and for different homoeology group. *H. chilense* and *H. vulgare* addition lines were kindly provided by Steve Reader, JIC, Norwich, UK. The presence of each *Hordeum* sp. chromosome in parental and F1 wheat lines used in this work was confirmed by both PCR assays previously described (Liu et al., 1996; Hagras et al., 2005) and *in situ* hybridization.

Seeds obtained from genetic crosses were germinated on wet filter paper in the dark for 5 days at 4°C, followed by a period of 24h at 25°C. Plants were then growth in the greenhouse at 26°C during the day and 18°C during the night (16 h photoperiod).

### Fluorescence *in situ* hybridization

Fluorescence genomic *in situ* hybridization (GISH) was used to study chromosome associations between *H. chilense* and *H. vulgare* chromosomes in the wheat background as described previously (Prieto et al., 2004b). Root tips were collected from germinating seeds and were pre-treated for 4 h in a 0.05% colchicine solution at 25°C and fixed in 100% ethanol-acetic acid, 3:1 (v/v), for at least a week at room temperature. Spikes in meiosis were collected from mature plants and preserved in 100% ethanol-acetic acid, 3:1 (v/v) until were used to characterize chromosome associations. Chromosome spreads were prepared from both root tips cells and pollen mother cells (PMCs) at meiosis. Root tips and anthers were macerated in a drop of 45% glacial acetic acid on ethanol-cleaned slides, squashed under a cover slip and dipped in liquid nitrogen in order to fix the plant material on the slide. The cover slip was removed and the slides were air-dried and stored at 4°C until used.

Both total genomic *H. vulgare* and *H. chilense* DNA were labeled by nick translation with biotin-11-(Boehringer Mannheim Biochemicals, Germany) and digoxigenin-11-dUTP (Roche Applied Science, Indianapolis, IN, USA), respectively, and used as probes. Both probes were mixed to a final concentration of 5 ng/μl in the hybridization mixture. The hybridization mixture consisted of 50% formamide, 2 × SCC, 5 ng of biotin-labeled or digoxigenin-labeled probe, 10% dextran sulfate, 0.14 μg of yeast tRNA, 0.1 μg of sonicated salmon sperm DNA and 0.005 μg of glycogen. Biotin-labeled *H. vulgare* DNA and digoxigenin-labeled *H. chilense* DNA were detected with a streptavidin- Cy3 conjugate (Sigma, St. Louis, MO, USA) and antidigoxigenin-FITC (Roche Diagnostics, Meylan, France), respectively. Chromosomes were counterstained with DAPI (4′, 6-diamidino-2-phenylindole) and mounted in Vectashield (Vector Laboratories, Burlingame, CA, USA).

Chromosome spreads from somatic cells and anthers of the F1 wheat lines were reprobed with the barley subtelomeric tandem repeat HvT01, which was obtained by amplification by the polymerase chain reaction (PCR) from genomic DNA from the barley cv. Betzes using primers made according to the published sequence (Belostotsky and Ananiev, 1990). PCR conditions were previously described by Prieto et al. (2004b). The PCR product corresponding to this barley satellite HvT01 probe was labeled with digoxigenin-11-dUTP, (Roche Applied Science, Indianapolis, IN, USA) by nick translation and detected with antidigoxigenin-FITC (Roche Diagnostics, Meylan, France). Meiosis metaphase samples were also reprobed to label centromeres using the RT sequence included in the barley centromeric BAC7 (Hudakova et al., 2001), amplified by PCR following the same conditions as the amplification of the CCS1 centromeric repeat (Aragón-Alcaide et al., 1996), labeled with biotin-11-(Boehringer Mannheim Biochemicals, Germany) and detected with the streptavidin- Cy3 conjugate (Sigma, St. Louis, MO, USA).

### Fluorescence microscopy and image processing

Hybridization signals were visualized using a Nikon Eclipse 80i epifluorescence microscope. Images were captured with a Nikon CCD camera using the Nikon 3.0 software (Nikon Instruments Europe BV, Amstelveen, The Netherlands) and processed with Photoshop 11.0.2 software (Adobe Systems Inc., San Jose, California, USA).

### Statistical analysis

Statistical analyses were performed using STATISTIX 10.0 software (Analytical Software, Tallahassee, FL, USA). Anaphase I combinations were evaluated by an analysis of variance (ANOVA) as a completely randomized design. This analysis included a tangent transformation in the anaphase I combination where only one pole of the meiocytes showed *H. chilense* and *H. vulgare* signals. Tetrad combinations were analyzed by the Kruskal–Wallis test (nonparametric one-way analysis of variance).

## Results

### Development of double monosomic *H. vulgare-H*. chilense addition lines in wheat

**Figure 1:**
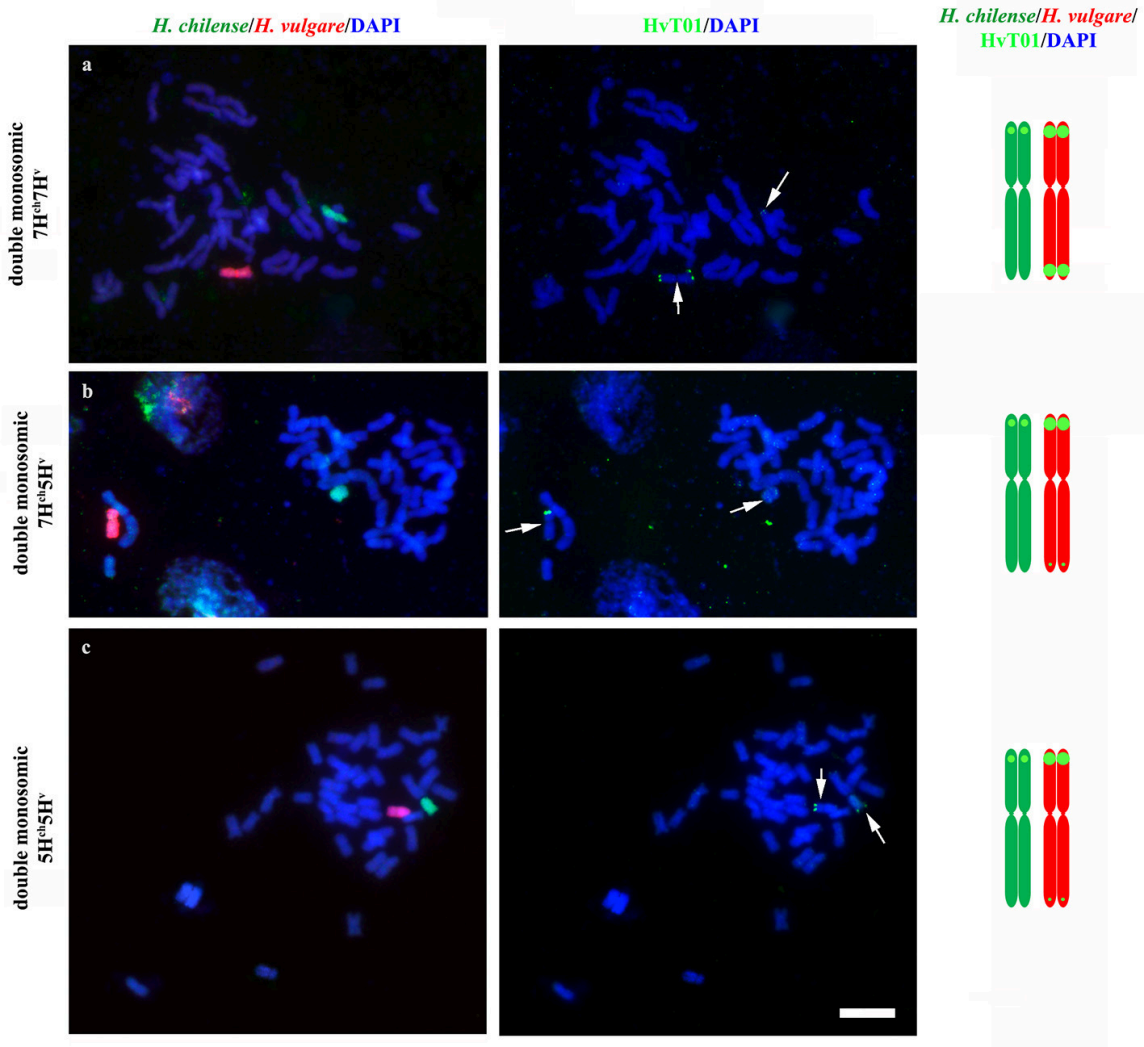
*Hordeum chilense* and *H. vulgare* double monosomic addition lines in wheat and physical location of the HvT01 subtelomeric repeat on barley chromosomes. *Hordeum chilense* (green) and *H. vulgare* (red) chromosomes were detected in GISH experiments in somatic chromosome spreads. In addition, the HvT01 subtelomeric sequence from barley was also detected (green). Total genomic DNA was counterstained with DAPI (blue). **(a)** Double monosomic 7H^ch^7H^v^ addition line, including a diagram showing the HvT01subtelomeric signals in all barley chromosome arms, except in 7H^ch^L arm. **(b)** Double monosomic 7H^ch^5H^v^ addition line, including a diagram showing the subtelomeric barley sequence in all barley chromosome arms except in 7H^ch^L arm. **(c)** Double monosomic 5H^ch^5H^v^ addition line, including a diagram showing the subtelomeric barley sequence in all barley chromosome arms, except in 5H^ch^L arm. Bar represents 10 μm.

Crosses between disomic *H. chilense* and *H. vulgare* addition lines in bread wheat carrying chromosomes 7H^ch^ and 7H^v^, respectively, were made to obtain double monosomic barley additions in wheat carrying homoeologous *H. chilense* and *H. vulgare* chromosome 7. Similarly, genetic crosses between disomic *H. chilense* and *H. vulgare* addition lines in bread wheat for chromosomes 7H^ch^ and 5H^v^, respectively, were made to obtain double monosomic wheat lines carrying non-homoeologous chromosomes 7H^ch^ and 5H^v^. Finally, to corroborate observations on chromosome associations in a different homoeology group, we also developed genetic crosses between disomic *H. chilense* and *H. vulgare* addition lines in bread wheat for chromosomes 5H^ch^ and 5H^v^, respectively, to obtain double monosomic barley additions in wheat lines carrying homoeologous *H. chilense* and *H. vulgare* chromosomes for group 5. The F_1_ hybrid progeny from each genetic cross was analyzed by GISH to ensure that they retained the expected both *H. chilense* and *H. vulgare* chromosomes (Figure 1). All the plants from all the genetic crosses carried both barley chromosomes. In addition, fluorescence *in situ* hybridization (FISH) was also performed in these wheat lines using the barley subtelomeric HvT01 repeat as a probe to label polymorphisms between the subtelomeric regions from the *H. chilense* and *H. vulgare* chromosomes added to the wheat background (Figure 1). Chromosome 7H^v^ had two strong signals for the barley subtelomeric HvT01 sequence on both chromosome arms meanwhile there was only a weaker signal on the short arm of chromosome 7H^ch^. Both 5H^ch^ and 5H^v^ chromosomes had a signal on the short arm for the HvT01 probe, which was stronger in the case of 5H^v^ chromosome, and only a weak signal on the subtelomeric region of the long arm of chromosome 5H^v^ was detected, which sometimes cannot be clearly seen and depended on the FISH experiment (Figure 1). No HvT01 subtelomeric signals were detected on the wheat chromosomes. The F1 progeny from each genetic cross was also growth until meiosis with the aim of studying the meiotic behavior of both *Hordeum* chromosomes by *in situ* hybridization in PMCs in the wheat background.

### Homoeologous wild and cultivated barley chromosomes can fully associate in pairs in wheat in the presence of the Ph1 locus

**Figure 2:**
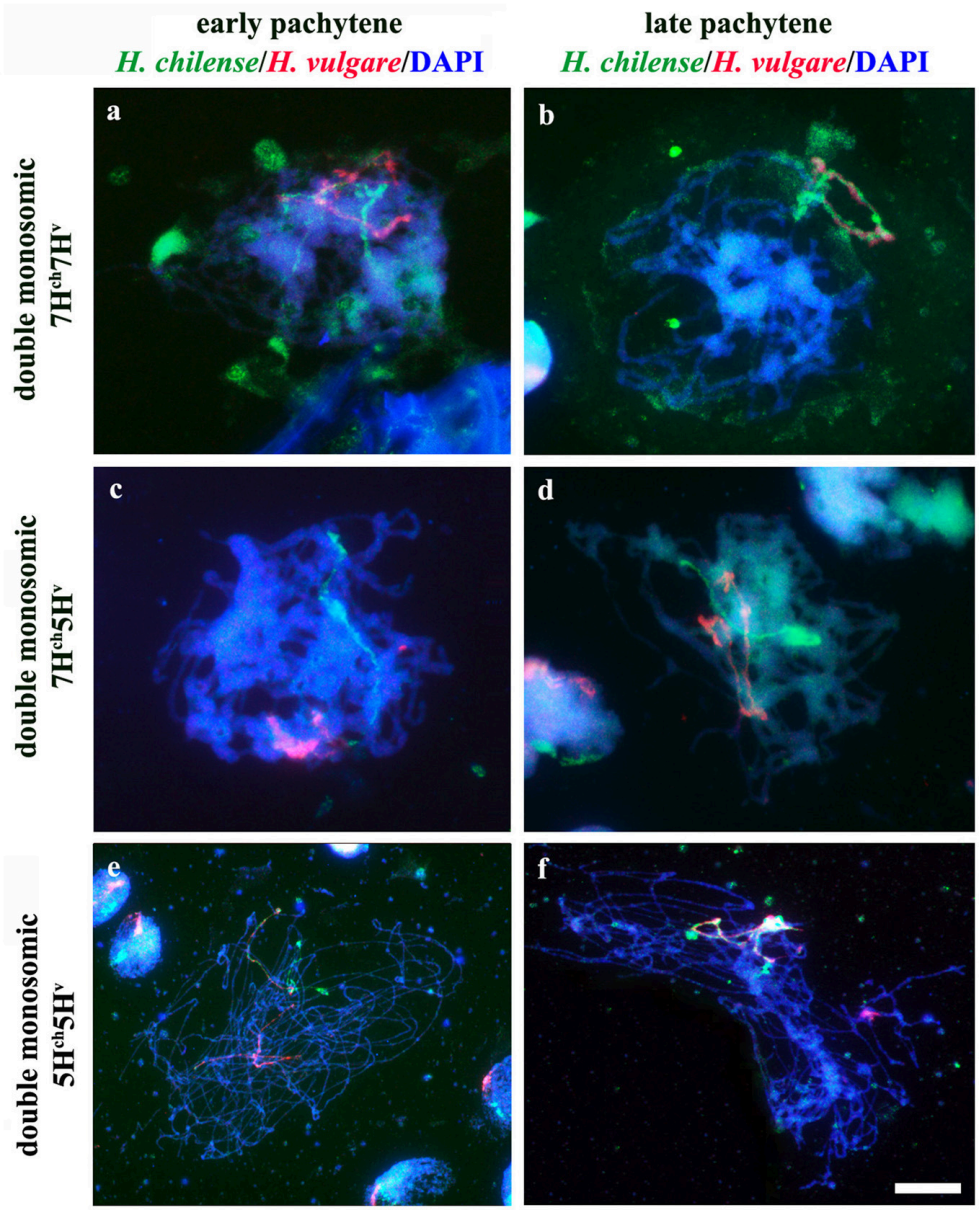
Behavior of homoeologous and non-homoeologous barley chromosomes during early meiosis in wheat. *Hordeum chilense* chromosomes are visualized in green and *H. vulgare chromosomes* are visualized in red. **(a)** Double monosomic 7H^ch^7H^v^ addition line showing both barley chromosomes un-associated at early pachytene. **(b)** Homoeologous barley chromosomes are fully associated at late pachytene in the double monosomic 7H^ch^7H^v^ addition line. **(c)** Double monosomic 7H^ch^5H^v^ addition line showing both barley chromosomes un-associated at early pachytene. **(d)** Non-homoeologous 7H^ch^ and 5H^v^ chromosomes remained un-associated at late pachytene. **(e)** Double monosomic 5H^ch^5H^v^ addition line showing homoeologous wild and cultivated barley chromosomes half-paired at early pachytene. **(f)** Double monosomic 5H^ch^5H^v^ addition line showing both homoeologous *Hordeum* chromosomes fully associated. Bar represents 10 μm.

Chromosome pairing was analyzed during early meiosis by GISH in F_1_ plants carrying one copy of *H. chilense* and one copy of *H. vulgare* homoeologous chromosomes (7H^ch^ and 7H^v^) and it was compared to those carrying non-homoeologous *H. chilense* and *H. vulgare* chromosomes (7H^ch^ and 5H^v^, respectively). Experiments were developed in around a 100 cells of each genomic combination in prophase I of meiosis. Both wild and cultivated barley chromosomes were visualized simultaneously in the wheat background (Figure 2). In both cases, *H. chilense* and *H. vulgare* chromosomes were in proximity in the nucleus in early prophase (Figures 2a,c). As meiosis progressed, GISH experiments showed homoeologous *H. chilense* and *H. vulgare* chromosomes always fully-associated in pairs along the whole chromosome (Figure 2b). In contrast, non-homoeologous *Hordeum* chromosomes 7H^ch^ and 5H^v^ were not observed associated at this meiotic stage at any time, remaining always un-associated (Figure 2d).

GISH experiments were also carried out in cells in prophase I of meiosis in F_1_ plants carrying homoeologous chromosomes from *H. chilense* and *H. vulgare* for another homoeology group (group 5), with the aim of confirming the observations on chromosome associations between homoeologous chromosomes 7H^ch^ and 7H^v^ in the wheat background. Results showed that homoeologous *Hordeum* chromosomes 5H^ch^ and 5H^v^ did also associate in pairs during early meiosis in the wheat background, even in the presence of the *Ph1* locus (Figures 2e,f), suggesting that chromosome pairing between homoeologous chromosomes from two different *Hordeum* species is not hampered by the *Ph1* locus. In addition, results suggested that homoeologous barley chromosomes shared enough similar DNA sequences to recognize each other, a conformational chromatin change is observed in both homoeologues and chromosomes are finally associated completely in pairs.

### Subtelomeres might hamper chromosome associations between non-homologous hordeum chromosomes in the wheat background

**Figure 3:**
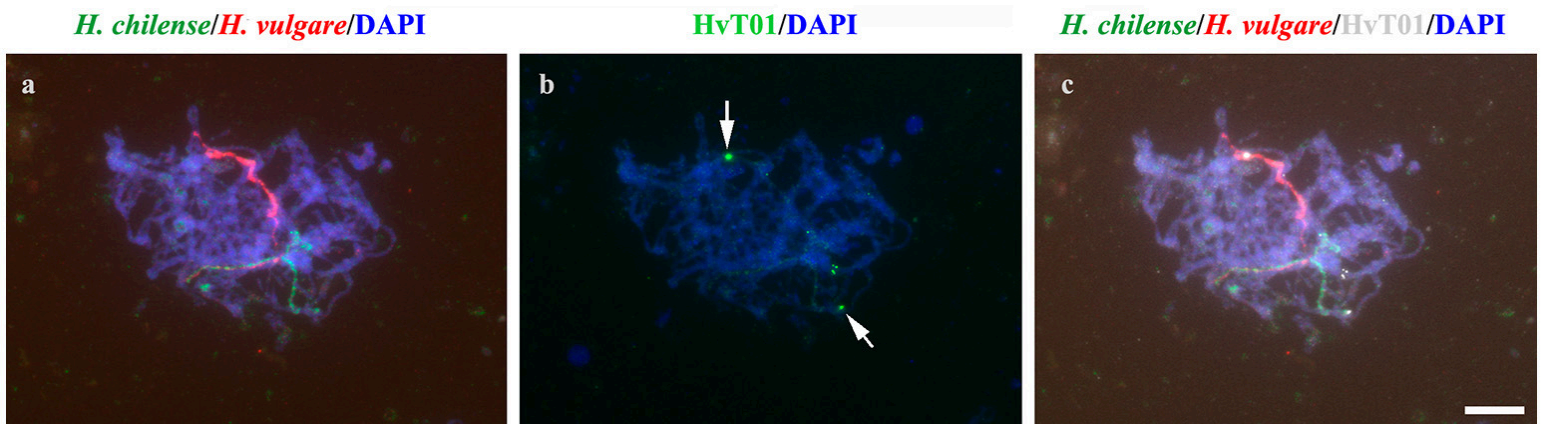
Detection of the subtelomeric HvT01 sequence in the wild and cultivated barley chromosomes in the 5H^ch^5H^v^ double monosomic addition line. DNA was counterstained with DAPI (blue). **(a)** GISH of both *H. chilense* (green) and *H. vulgare* (red) homoeologous chromosomes initiating pairing during early pachytene. **(b)** Detection of the subtelomeric HvT01 probe (green) on the same cell. **(c)** Merge image showing the subtelomeric HvT01 signal (overlay in white) on the terminal region of the unpaired homoeologous wild and cultivated barley chromosome arms (arrowed). Bar represents 10 μm.

We have described that in the absence of homologous chromosomes, wild and cultivated barley homoeologous chromosomes can still recognize each other and associate completely in pairs during early meiosis. In addition, we have observed that, in these cases, initial chromosome recognition occurred by the chromosome ends where none or little copy number of the subtelomeric HvT01 repeat were detected, i.e., the long arm of homoeologous chromosomes 5H^ch^ and 5H^v^ (Figure 3). Thus, homoeologous *Hordeum* chromosomes did recognize and associate in pairs by these chromosome ends, which made chromosome recognition less restrictive and allowed homoeologous to recognize and associate in pairs. These observations were similar in the cells detected at the same stage from the double monosomic 7H^ch^7H^v^ addition line in wheat (data not shown). Moreover, the observations were consistent in all the cells detected at the initiation of pairing (26 and 18 cells from the double monosomic 5H^ch^5H^v^ and 7H^ch^7H^v^ addition lines in wheat, respectively). Once homoeologous chromosomes had associated by these chromosome ends, a conformational change was observed along both homoeologous *Hordeum* chromosomes and pairing progressed along both chromosomes allowing a complete chromosome association between them (Figure 3).

### Crossing over does not occur between wild and cultivated barley chromosomes in wheat although they previously associated in early meiosis

**Figure 4:**
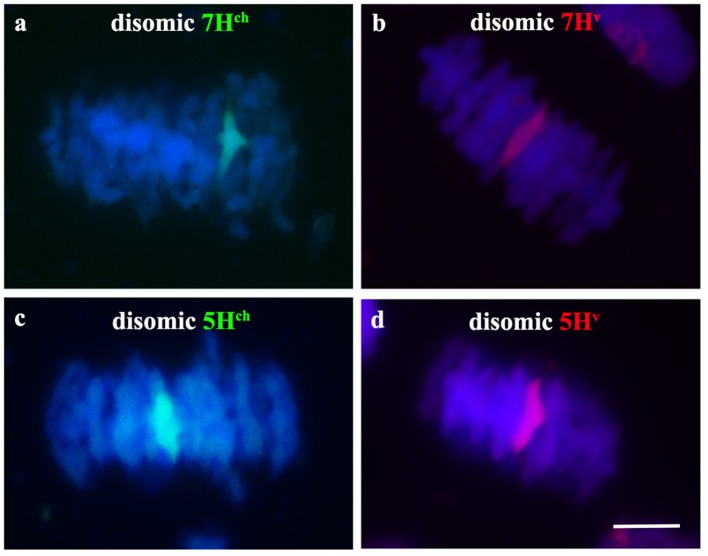
*Hordeum chilense* and *H. vulgare* chromosome behavior during metaphase I in parental *H. chilense* and *H. vulgare* disosomic addition lines in wheat. *Hordeum chilense* (green) and *H. vulgare* (red) chromosomes were observed always associated in pairs in all the cells in metaphase I in each *H. chilense* and *H. vulgare* disomic addition line. DNA was counterstained with DAPI (blue). **(a)** *H. chilense* chromosome 7H^ch^ disomic addition line. **(b)** *H. vulgare* chromosome 7H^v^ disomic addition line. **(c)** *H. chilense* chromosome 5H^ch^ disomic addition line. **(d)** *H. vulgare* chromosome 5H^v^ disomic addition line. Bar represents 10 μm.

**Figure 5:**
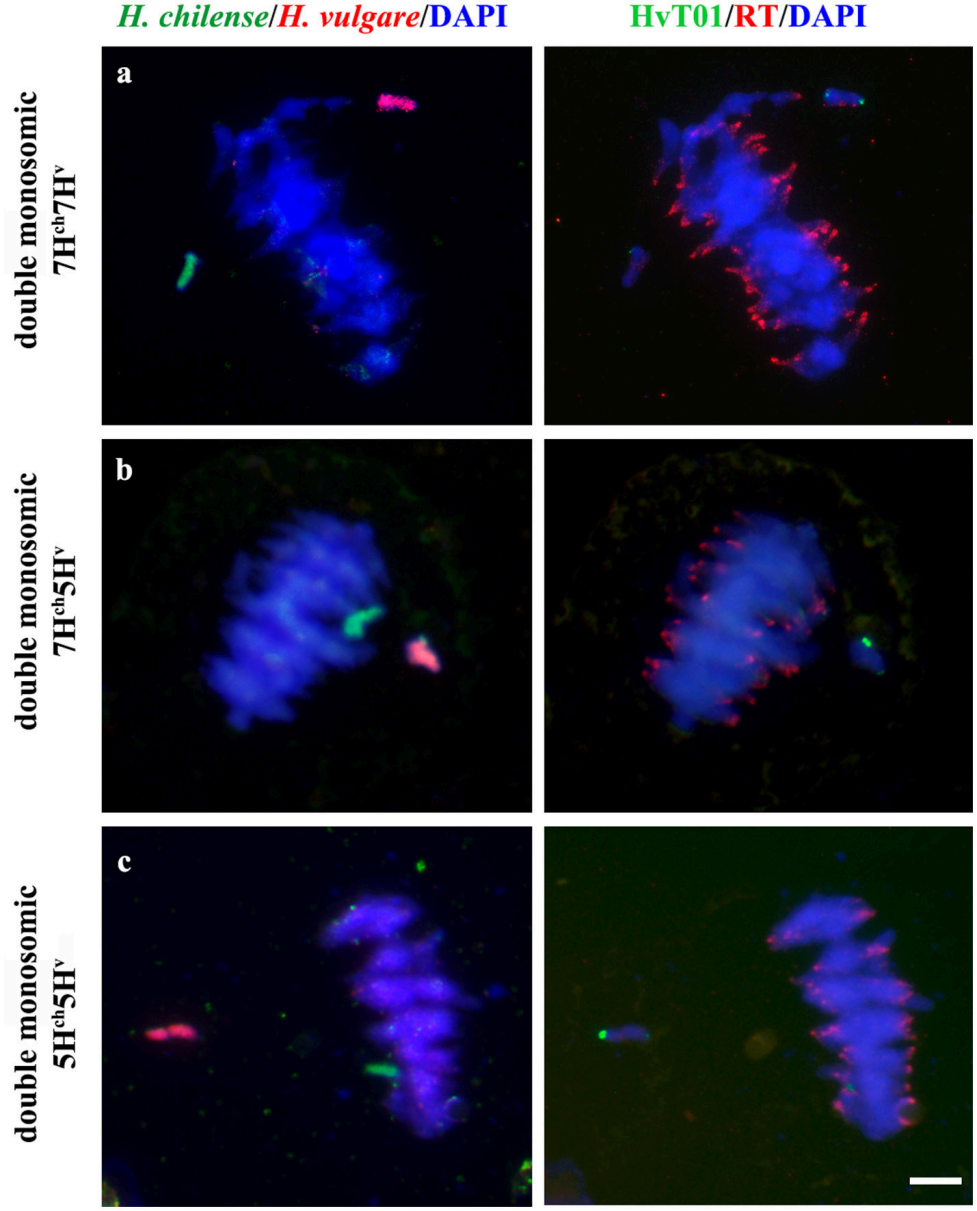
*Hordeum chilense* and *H. vulgare* chromosome behavior in double monosomic barley addition lines in wheat during metaphase I. *Hordeum chilense* (green) and *H. vulgare* (red) chromosomes remained unassociated in all the cases. DNA was counterstained with DAPI (blue). Centromeres (red) were labeled with RT sequence to show the correct orientation of wheat chromosomes during metaphase I. Subtelomeres on barley chromosomes were labeled in green. **(a)** Double monosomic 7H^ch^7H^v^ addition line. **(b)** Double monosomic 7H^ch^5H^v^ addition line. **(c)** Double monosomic 5H^ch^5H^v^ addition line. Bar represents 10 μm.

Once we observed full chromosome associations between homoeologous *Hordeum* chromosomes during early meiosis in the wheat background, we also analyzed chromosome behavior of both wild and cultivated barley chromosomes during metaphase I of meiosis in PMCs from double monosomic *H. chilense* and *H. vulgare* additions in wheat lines, both for the same and different homoeologous chromosomes. Meiosis metaphase I was also checked in the disomic *H. chilense* and *H. vulgare* addition lines in wheat used as parental lines for the genetic crosses developed in this work, to obtain the double *H. chilense* and *H. vulgare* double monosomic addition lines. Chromosome stability of the parental lines was confirmed as the homologous barley chromosomes carried in the *H. chilense* and *H. vulgare* disomic addition lines were always observed associated in pairs, indicating that crossing over occurred between homologous barley chromosomes (Figure 4). Similarly, wheat chromosomes associated correctly in bivalents at meiosis metaphase I and orientated by centromeres properly in double monosomic *H. chilense* and *H. vulgare* additions in wheat lines, both for the same and different homoeologous chromosomes (Figure 5). In contrast, *H. chilense* and *H. vulgare* chromosomes remained always un-associated in all the cells analyzed for the three different genetic combinations analyzed (Figure 5), despite the fact that homoeologous *H. chilense* and *H. vulgare* chromosomes (7H^ch^7H^v^ and 5H^ch^5H^v^, respectively) did completely associate in pairs in early meiosis. These observations suggested that, although wild and cultivated barley homoeologous chromosomes can fully associate during pachytene, crossing over did not occur later between these chromosomes. Consequently, *Hordeum* homoeologous chromosomes were never observed associated by chiasmata during metaphase I and always remained as univalent (Figure 5), suggesting other requirements for crossing over rather than full previous chromosome associations or similarities in the DNA sequence.

### Chromosome segregation does not depend on previous chromosome associations during early meiosis

**Figure 6:**
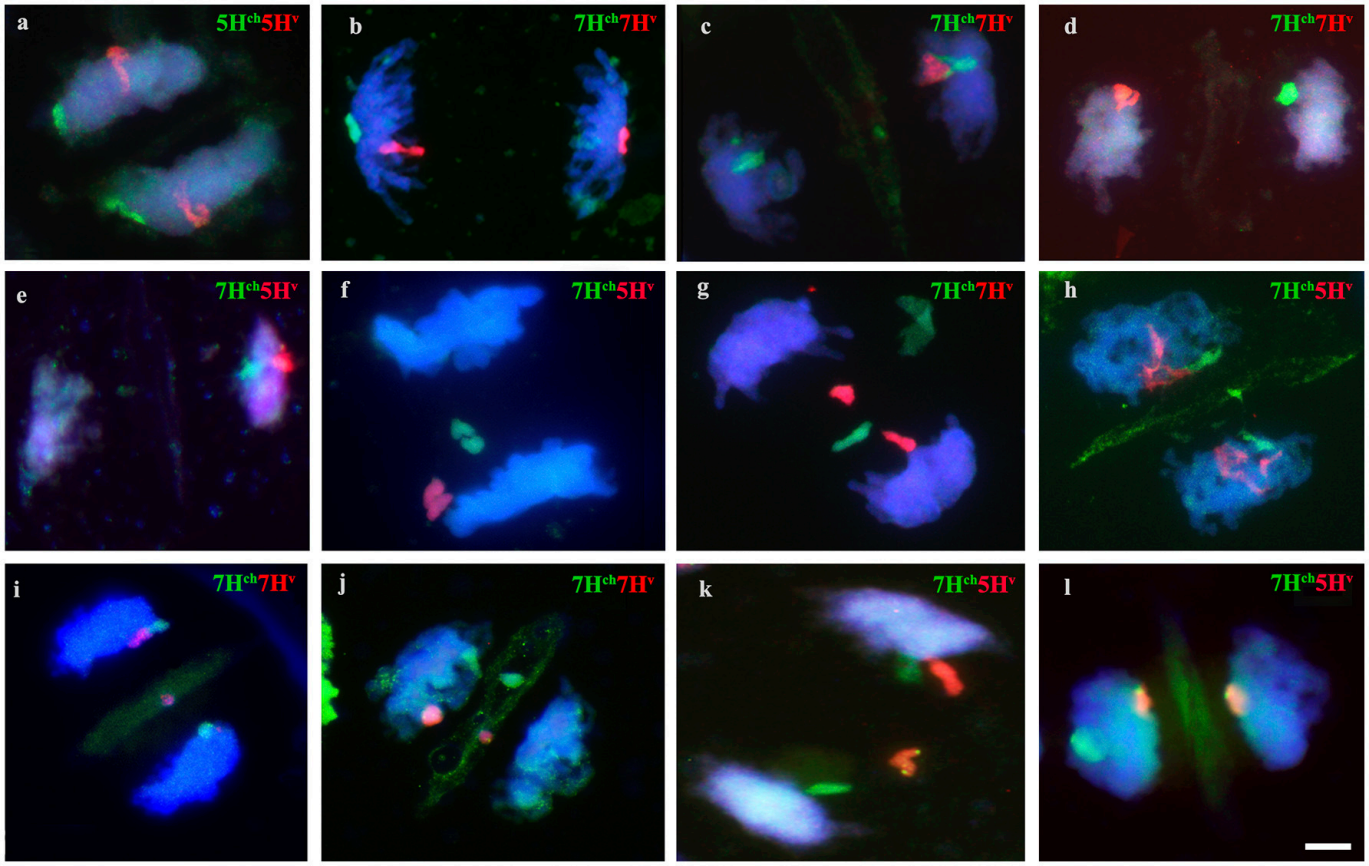
Behavior of *Hordeum chilense* and *H. vulgare* chromosomes in double monosomic barley addition lines in wheat during anaphase I of meiosis. Examples of barley chromosome segregation after metaphase I. *Hordeum chilense* and *H. vulgare* were visualized in green and red, respectively. DNA was counterstained with DAPI (blue). **(a)** Double monosomic 5H^ch^5H^v^ addition line, **(b)** Double monosomic 7H^ch^7H^v^ addition line, **(c)** Double monosomic 7H^ch^7H^v^ addition line; **(d)** Double monosomic 7H^ch^7H^v^ addition line. **(e)** Double monosomic 7H^ch^5H^v^ addition line. **(f)** Both 7H^ch^5H^v^ barley chromosomes remained delayed. **(g)** Both 7H^ch^7H^v^ barley chromosomes remained delayed and a misdivision of chromosome 7H^v^ was also observed. One or both barley micronuclei were positioned in the equatorial region on telophase I in **(h)** Double monosomic 7H^ch^5H^v^ addition line, **(i)** Double monosomic 7H^ch^7H^v^ addition line, and **(j)** Double monosomic 7H^ch^7H^v^ addition line. The subtelomeric HvT01 probe was used in GISH experiments performed in cells in telophase I from the double monosomic 7H^ch^5H^v^ addition line to visualize **(k)** 5H^v^ chromosome misdivision or **(l)** 5H^v^ chromosome segregation. Bar represents 10 μm.

Each PMC analyzed at the MI stage was characterized by the presence of two barley univalents in double monosomic *H. chilense-H. vulgare* addition lines. Around 300 cells were observed in meiosis anaphase I. GISH analysis showed that both wild and cultivated barley univalents segregated simultaneously with wheat bivalents at stage anaphase I (Figure 6). All the different possible situations for chromosome segregation of the unpaired *H. chilense* and *H. vulgare* chromosomes were identified (Figures 6a–e, Table 1): (i) both barley chromosome were detected in both nuclei; (ii) only *H. vulgare* chromosome was detected in both nuclei; (iii) only *H. chilense* chromosome was detected in both nuclei; (iv) each barley chromosome was detected in each daughter nucleus; and (v) both barley chromosomes were detected in the same anaphase/telophase pole. These different situations for chromosome segregation of both barley chromosomes were found in all the genetic combinations (7H^ch^7H^v^, 5H^ch^5H^v^, and 7H^ch^5H^v^ double monosomic addition lines) in the wheat background, although the ratio between them varied depending on the genetic stock (Table 1). Nevertheless, no significant differences were found for *H. chilense* and *H. vulgare* chromosome segregation between the different genetic combinations (Table 1), despite the fact that the most frequent observation for the segregation of the wild and cultivated barley chromosomes was different in 7H^ch^7H^v^ addition line compared to 5H^ch^5H^v^, and 7H^ch^5H^v^ addition lines (Table 1). Moreover, no significant differences were found on the behavior of the *Hordeum* chromosomes within the same F1 line. These results suggest that chromosome segregation in double *H. chilense* and *H. vulgare* monosomic addition lines in wheat occurred randomly, regardless the barley chromosomes added to the wheat background and whether or not chromosome associations took place previously in early meiosis.

**Table 1:** **(A)** Total number of PMCs scored at anaphase I showing the different combinations observed for both *Hordeum* chromosomes added to the wheat background. The most frequent observation per line and the total number of meiocytes examined are shown in bold. **(B)** Quantification of meiocytes (%) for each observation.

**(A)**						
**Wheat lines**	**Number of PMCs scored in Anaphase I**					
	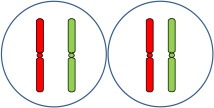	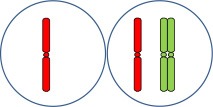	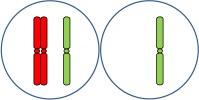	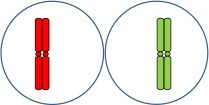	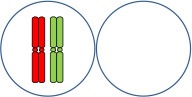	**Total**
7H^ch^ 7H^v^ addition	23	**49**	9	35	12	128
7H^ch^5H^v^ addition	**24**	19	12	6	16	77
5H^ch^5H^v^ addition	**35**	22	5	14	10	86
**Total**	82	90	26	55	38	**291**
**(B)**						
	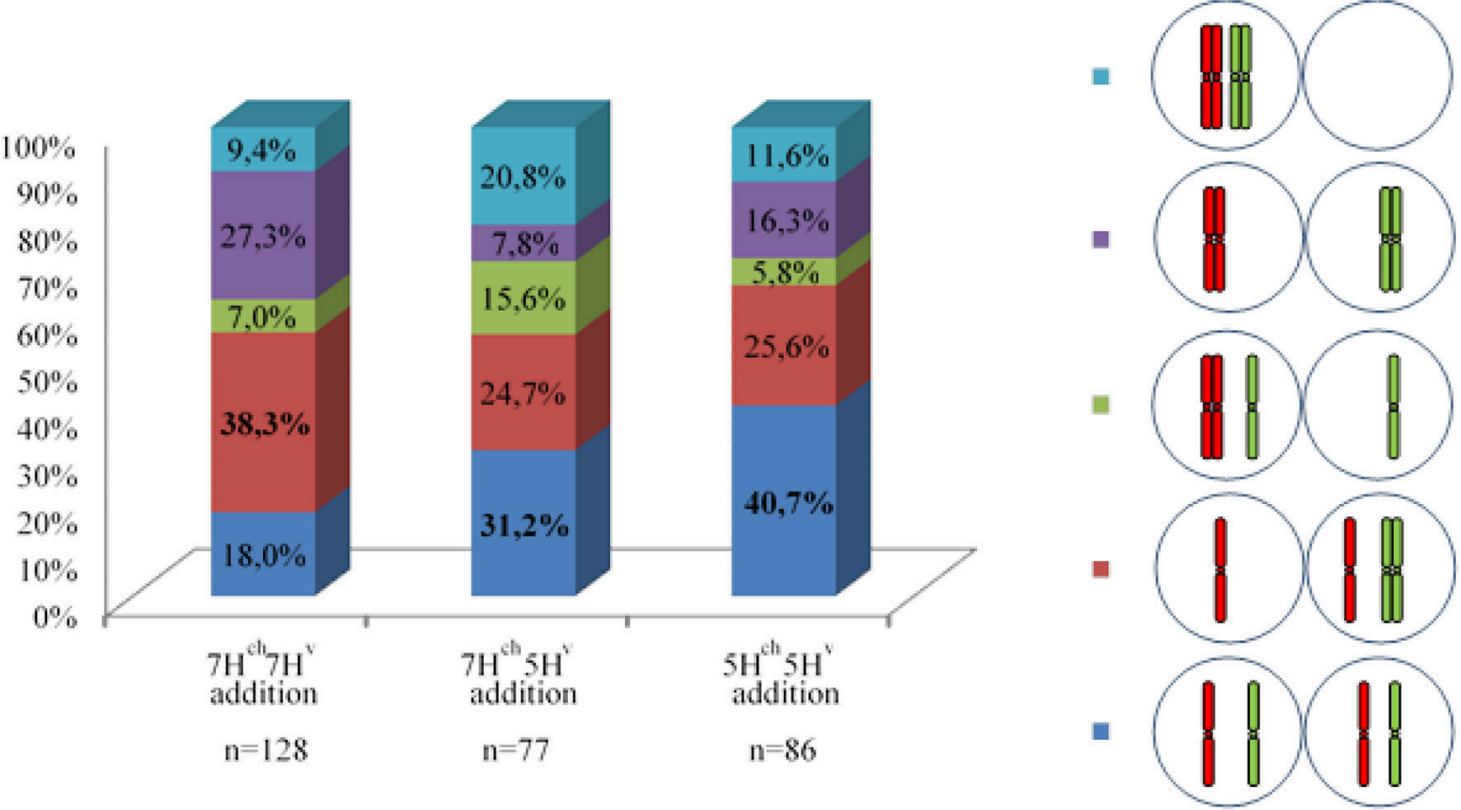					

Chromosomes delay was usually observed in double monosomic *H. chilense* and *H. vulgare* addition lines in late anaphase I/telophase I (Figures 6f,g), and the presence of chromatin across the equator during phragmoplast formation either from *H. chilense, H. vulgare* or both species was also observed (Figures 6h–j). Missegregation or chromosome breaks that occurred in anaphase I in the double monosomic *H. chilense* and *H. vulgare* addition lines cannot be distinguished from sister chromatids segregation unless using, among others, the HvT01 subtelomeric probe (Figures 6k,l).

**Figure 7:**
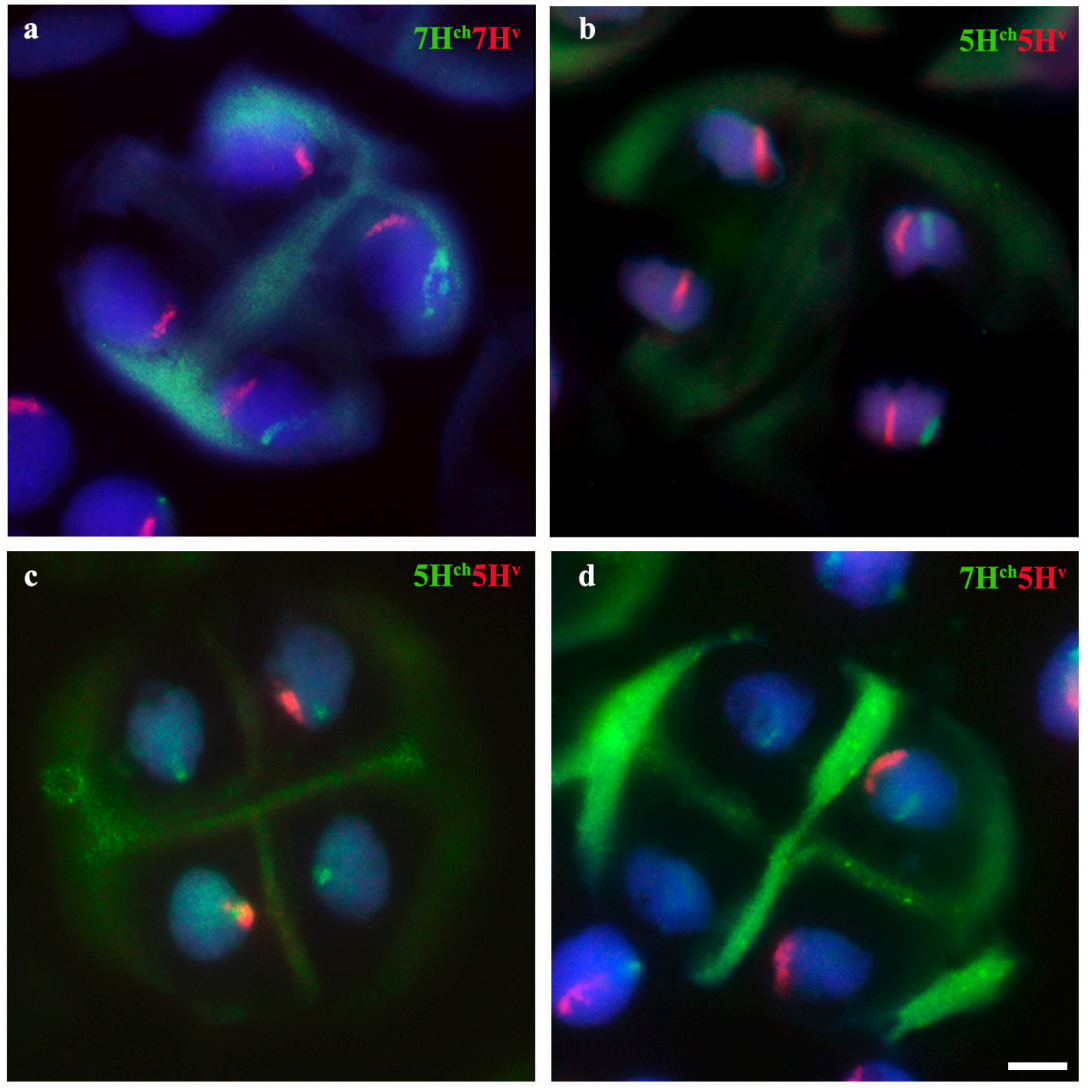
Behavior of *H. chilense* and *H. vulgare* chromosomes in double monosomic *Hordeum* addition lines in wheat after the second meiosis division. Examples of the most frequent observations for both *H. chilense* (green) and *H. vulgare* (red) chromosomes are shown. DNA was counterstained with DAPI (blue). **(a)** *Hordeum vulgare* and *H. chilense* chromatin detected in four and two PMCs, respectively, in the double monosomic 7H^ch^7H^v^ wheat line. **(b)** *Hordeum vulgare* and *H. chilense* chromatin detected in four and in two PMCs, respectively, in the double monosomic 5H^ch^5H^v^ wheat line. **(c)** Four *H. chilense* signals and two *H. vulgare* signals were observed in the double monosomic 5H^ch^5H^v^ wheat line at the same frequency as reported in **b**. **(d)** Wild and cultivated barley signals are present in the same nucleus and only one each in another two different nucleus. Bar represents 10 μm.

Depending on chromosome segregation of both wild and cultivated barley univalents during meiosis I, the number of different genetic combinations in the PMC increased during MII, resulting in a wide range of meiotic phenotypes observed (nineteen different cases; Table 2). The most frequent *H. chilense* and *H. vulgare* chromosome combination observed for each genetic combination during telophase II was different depending on whether *H. chilense* and *H. vulgare* chromosomes were included in the same or in different homoeology group (Figure 7; Table 2), but no significant differences were found. Results suggested that both sister chromatids separation and misdivision did occur randomly independently of the barley chromosome combination.

**Table 2:** **(A)** Number of meiocytes showing the different combinations for both *Hordeum* chromosomes added to the wheat background. The most frequent observation per line and the total number of PMCs examined are shown in bold. **(B)** Quatification of meiocytes (%) for each observation.

**(A)**																				
**Wheat lines**	**Number of PMCs scored in tetrads**																			
	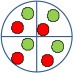	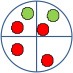	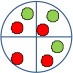	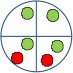	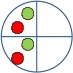	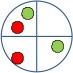	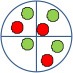	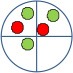	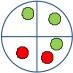	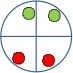	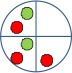	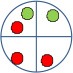	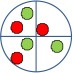	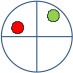	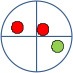	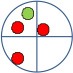	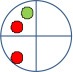	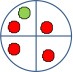	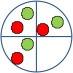	**Total**
7H^ch^7H^v^ addition	2	**6**	5	1	0	1	3	0	0	0	0	1	1	1	1	2	1	1	0	26
7H^ch^5H^v^ addition	1	1	0	2	2	**5**	0	0	0	3	1	1	0	0	0	0	3	0	3	22
5H^ch^5H^v^ addition	4	**10**	2	**10**	7	2	0	1	0	3	3	0	0	0	0	1	0	0	0	43
Total	7	17	7	13	9	8	3	1	0	6	4	2	1	1	1	3	4	1	3	**91**
**(B)**																				
	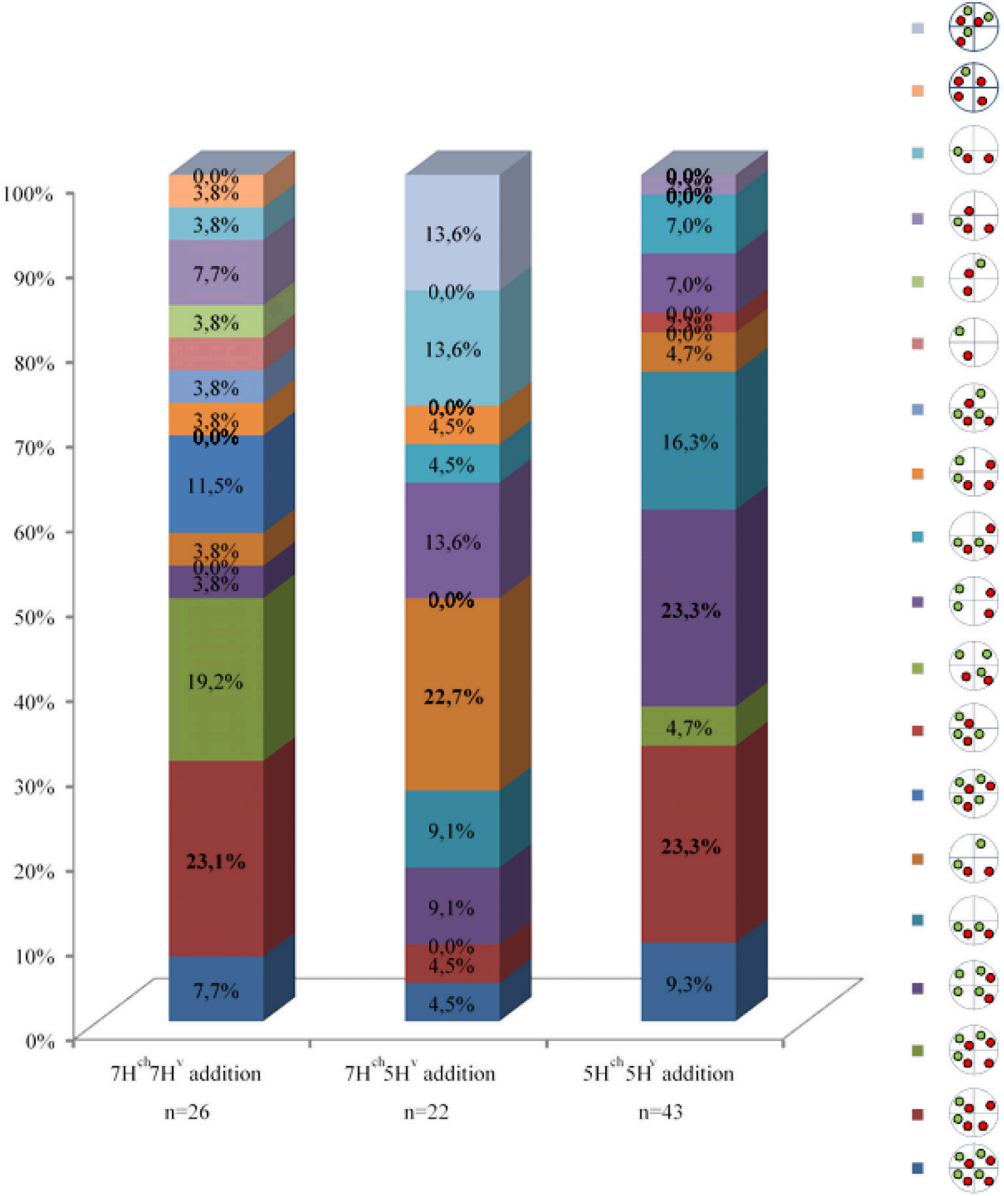																			

## Discussion

Little is known about how chromosomes recognize each other to correctly associate in pairs at early meiosis and recombine. This is a key question for plant breeders to transfer genetic variability from related species into a crop like wheat. The lack of recombination between cultivated wheat and alien chromosomes limits the transfer of novel traits from relatives to wheat because *Ph1* suppresses homoeologous recombination between wheat and related species (Riley and Chapman, 1958; Sears, 1976). Different meiosis studies on chromosome pairing have been developed using wheat lines carrying an addition of one pair of homologous chromosomes or chromosome segments from one related species into wheat (Mikhailova et al., 1998; Maestra et al., 2002; Prieto et al., 2004a) or hybrids between wheat and relatives (Molnár-Láng et al., 2014; Rey et al., 2017). In this work, we have developed wheat lines carrying double monosomic chromosome additions for wild and cultivated barley for the same and for different homoeology group, respectively, which allowed us to track simultaneously by GISH a couple of extra homoeologous and non-homoeologous chromosomes from two different *Hordeum* species during early meiosis. These double monosomic addition lines can contribute to go deeper into the knowledge of how chromosomes recognize and associate in pairs in the wheat background in the presence of the *Ph1* locus. In addition, most of the works carrying alien chromosomes in the wheat background are focused in meiosis metaphase I or later stages (Molnár-Láng et al., 2000; Silkova et al., 2014). The analysis of chromosome pairing focused only in metaphase I can result in an underestimation of homoeologous associations that might occur earlier in meiosis, as chromosomes might remain mostly as univalents due to the lack of homoeologous recombination. Few works analyzed the behavior of an extra pair of chromosomes at early stages of meiosis (Aragón-Alcaide et al., 1997; Prieto et al., 2004a; Valenzuela et al., 2012, 2013; Koo et al., 2016). Homologous barley chromosomes have been previously observed associated during early meiosis and metaphase I in disomic addition lines in wheat (Aragón-Alcaide et al., 1997; Calderón et al., 2014). In this study we have reported by GISH analysis that homoeologous wild and cultivated barley chromosomes can also fully associate in pairs in the wheat background during early meiosis and that such chromosome pairing occurred even in the presence of the *Ph1* locus, although homoeologous barley chromosomes did not cross over and were always observed as univalent in metaphase I. The role of the *Ph1* locus was recently narrowed down preventing recombination between related chromosomes in interspecific hybrids (Moore, 2014; Martín et al., 2017). Our results clearly showed that the *Ph1* locus does not hamper homoeologous chromosome associations but crossing over. Nevertheless, homoeologous recombination between related *Aegilops geniculata* and *Ae. searsii* has been detected in the wheat background in the presence of the *Ph1* locus due to the presence of chromosome 5 Mg of *Ae. geniculata*, which harbors a homoeologous recombination promoter factor (Koo et al., 2016). Recombination frequencies between *H. vulgare* and *H. bulbosum* homoeologues have been previously detected but are lower than association frequencies (Zhang et al., 1999), probably because of non-chiasmate associations (Orellana, 1985). Homologous pairing has been also described in the absence of synapsis and meiotic recombination in *Caenorhabditis elegans* (Dernburg et al., 1998). Our results showed that homoeologous *H. chilense* and *H. vulgare* chromosomes associated in pairs in wheat in the absence of crossing over and that the *Ph1* locus does not prevent such chromosome recognition and association between homoeologues. It is also worthy to mention that, although *H. chilense* and *H. vulgare* are phylogenetically quite distant, even included in two different sections among the *Hordeum* genus (Blattner, 2009), both species share a high degree of similarities at the chromosomal level, as it has been reported between them and other species within this genus (Hernández et al., 2001; Aliyeva-Schnorr et al., 2016). Thus, other elements such as cohesins or the DNA sequence itself might play a major role on chromosome recognition and pairing at the onset of meiosis in a polyploidy like wheat.

So far, it is unclear whether initial recognition is mediated through protein-protein interactions, DNA base-pairing, or other chromosomal features. For example, a noncoding RNA (meiRNA-L) is responsible for the recombination-independent pairing of homologous loci in *Schizosaccharomyces pombe* (Ding et al., 2012). Other chromosomal features different from DNA/DNA recombinational interactions or RNA-mediated pairing have been proposed to be involved in the homologous recognition such as the pattern of cohesins distribution in the axial elements of unmatched meiotic chromosomes in mice and *S. pombe* (Ishiguro et al., 2011; Ding et al., 2016). Subtelomeres have been also reported as crucial to promote chromosome recognition and pairing between homologous chromosomes (González-García et al., 2006; Calderón et al., 2014). In our study, variability for the HvT01 subtelomeric sequence was found between *H. chilense* and *H. vulgare* chromosomes 5 and 7, particularly for the long arm of both chromosomes. We observed that although homoeologous chromosomes can potentially associate by the telomeres, subtelomeric DNA blocks might hamper homoeologous chromosome to correctly associate in pairs and thus, in the absence of homologs, chromosome recognition and association between homoeologues can occurred by the chromosome end where the subtelomeric repeats are shorter or absent. However, as meiosis progressed, the pairing signal initiated at these chromosome ends can be propagated along the whole chromosome, so that the homoeologues became fully associated by late pachytene. Thus, our results might suggest that subtelomeres can play a key role in the specificity of chromosome recognition, restricting chromosome recognition to true homologs and therefore hampering homoeologous chromosomes to recognize each other and associate. The implication was that DNA sequence(s) within the subtelomeric region must be important for the process of initial homolog recognition and pairing, although further studies are required to reveal how subtelomeres take part in such important meiosis processes.

The peculiarities of univalent behavior in meiosis have been extensively studied in wheat aneuploids, particularly for the relation between the means of a chromosome segregation and its inclusion into a microspore (Sears, 1952; Marais and Marais, 1994; Friebe et al., 2005; Lukaszewski, 2010). The knowledge about univalent behavior in meiosis is necessary for the directed development of wheat lines carrying alien introgressions since univalent are subjected of incorrect division and segregation. Thus, abnormities in meiosis result in various modifications and/or in the loss of a transferred chromosome (Silkova et al., 2014). Univalents in meiosis have a tendency to misdivide (break) across their centromeres producing telocentric. This process has been deeply described in wheat (Sears, 1952; Steinitz-Sears, 1966; Friebe et al., 2005), and used to generate different cytogenetic stocks (Sears and Sears, 1978; Lukaszewski, 1993, 1997). The most common alien introgression in wheat, chromosome translocations, is the result of centric misdivision and fusion of misdivision products. Translocations between *H. chilense* and *H. vulgare* have been detected previously when the genomes of these species are in the same background (Prieto et al., 2001). Our results overview the univalent behavior of two homoeologous and non-homoeologous barley chromosomes in the wheat background. We observed that chromosome misdivisions and sister chromatids segregated randomly at anaphase I similarly to previous works (Friebe et al., 2005), and independently of whether or not related chromosomes associate in pairs in early meiosis.

In summary, homoeologous wild and cultivated barley chromosomes were observed fully associated in pairs in early meiosis in the presence of the *Ph1* although crossing over did not occur at any time, as both chromosomes were always visualized as univalents during metaphase I. Whether or not homoeologous *Hordeum* chromosomes can crossover in the absence of the *Ph1* locus remains to be elucidated. In addition, the role of the terminal chromosome regions in chromosome recognition and paring and the proteins interacting with these chromosomes ends will be key questions to shed light in future works.

## Human and animal rights and informed consent

This article does not contain any studies with human participants or animals.

## Authors contributions

All authors contributed to this manuscript. MC and PP designed the research and performed the experiments. MC, MR, AM, and PP analyzed, discussed the results. PP and MC wrote the manuscript. All authors read and approved the manuscript.

### Conflict of interest statement

The authors declare that the research was conducted in the absence of any commercial or financial relationships that could be construed as a potential conflict of interest.
